# A Microphysiological Model to Mimic the Placental Remodeling during Early Stage of Pregnancy under Hypoxia-Induced Trophoblast Invasion

**DOI:** 10.3390/biomimetics9050289

**Published:** 2024-05-12

**Authors:** Seorin Jeong, Ahmed Fuwad, Sunhee Yoon, Tae-Joon Jeon, Sun Min Kim

**Affiliations:** 1 Department of Mechanical Engineering, Inha University, 100, Inha-ro, Michuhol-gu, Incheon 22212, Republic of Korea; tjfls0923@gmail.com (S.J.); ahmedsunny41@gmail.com (A.F.); 2 Department of Biomedical Engineering, School of Mechanical & Manufacturing Engineering (SMME), National University of Science and Technology (NUST), Islamabad 44000, Pakistan; 3 Department of Biological Sciences and Bioengineering, Inha University, 100, Inha-ro, Michuhol-gu, Incheon 22212, Republic of Korea; yoonsh0912@inha.ac.kr; 4 Biohybrid Systems Research Center, Inha University, 100, Inha-ro, Michuhol-gu, Incheon 22212, Republic of Korea; 5 Department of Biological Engineering, Inha University, 100, Inha-ro, Michuhol-gu, Incheon 22212, Republic of Korea

**Keywords:** HUVEC vessel remodeling, hypoxia, trophoblast cell invasion, 3D co-culture chip, preeclampsia, placenta

## Abstract

Placental trophoblast invasion is critical for establishing the maternal–fetal interface, yet the mechanisms driving trophoblast-induced maternal arterial remodeling remain elusive. To address this gap, we developed a three-dimensional microfluidic placenta-on-chip model that mimics early pregnancy placentation in a hypoxic environment. By studying human umbilical vein endothelial cells (HUVECs) under oxygen-deprived conditions upon trophoblast invasion, we observed significant HUVEC artery remodeling, suggesting the critical role of hypoxia in placentation. In particular, we found that trophoblasts secrete matrix metalloproteinase (MMP) proteins under hypoxic conditions, which contribute to arterial remodeling by the degradation of extracellular matrix components. This MMP-mediated remodeling is critical for facilitating trophoblast invasion and proper establishment of the maternal–fetal interface. In addition, our platform allows real-time monitoring of HUVEC vessel contraction during trophoblast interaction, providing valuable insights into the dynamic interplay between trophoblasts and maternal vasculature. Collectively, our findings highlight the importance of MMP-mediated arterial remodeling in placental development and underscore the potential of our platform to study pregnancy-related complications and evaluate therapeutic interventions.

## 1. Introduction

Preeclampsia is a serious hypertensive disorder of pregnancy that affects 2–8% of all pregnancies worldwide [1,2,3,4]. It is characterized by high blood pressure (hypertension) and proteinuria after 20 weeks of gestation and is a leading cause of maternal and fetal morbidity and mortality [5,6]. The exact etiology of preeclampsia is not fully understood, but recent studies suggest that oxygen regulation plays a critical role in its pathogenesis [7]. During pregnancy, the placenta plays a critical role in fetal development by regulating the exchange of oxygen, nutrients, and waste product exchange between the fetus and the mother [8]. Oxygen tension is tightly regulated by the placenta, and any disruption in this delicate balance can lead to adverse pregnancy outcomes, including preeclampsia, oxidative stress, placental disorders, and endothelial dysfunction [9].

Despite its pivotal importance, the placenta is a poorly understood organ, mainly due to the limitations of the conventional experimental techniques (Supplementary Table S1). The ex vivo studies based on the placenta tissue obtained after delivery could not simulate pregnancy pathologies and therefore provide limited insights [10,11]. Studies based on animal models such as guinea pig [12] or sheep [13] have the advantage of being able to obtain longitudinal samples unlike with ex vivo models. However, they are expensive and can only provide a limited understanding of placental physiology, which is different from human placenta physiology. Recently, some in vitro studies based on 2D platforms such as culture dishes or Transwells have been used to understand the placental mechanism. The 2D platforms are based on human-derived cells, which has the advantage of enabling physiological studies such as cell–cell interaction studies or signal pathways while simulating the human placental environment, but these models fail to replicate the three-dimensional (3D) structure of the placental barrier and complex physiological phenomenon, thus providing limited information [14,15,16,17,18].

More recently, with the advancement of micro-nanotechnology and molecular biology, microfluidic technology has been applied to the study of complex physiological processes, allowing for mimicry of complex human physiology at the microscale with high tunability as well as easy handling in terms of cell manipulation, recapitulation of complex 3D structures, and integration with external control and flow systems [19,20,21,22,23,24,25]. Recently, Blundell et al. used the microfluidic platform to mimic the placental barrier and analyze the transport of a drug (glyburide) from mother to fetus [26]. Similarly, in another study by the same group, the nutrient transport across the placental barrier was analyzed by fabricating a physiological barrier for glucose transport [27]. Furthermore, Lee et al. suggested a microfluidic platform to understand the transport barrier physiology of the human placenta by fabricating a placenta-on-a-chip that enables compartmentalized perfusion across a thin extracellular barrier; however, the results showed significantly lower shear stress levels compared to fetal capillaries, and the primary cells from the placenta need to be analyzed [28]. In addition, this chip only mimics the early stage of pregnancy, which excludes the effect of complex pregnancy factors such as oxygenation, hormones, and immune regulators [5]. More recently, Pu et al. utilized the commercially available 3D microfluidic chip to analyze the interaction between primary placental trophoblast cells and HUVECs under a chemical gradient condition. Under the dynamic flow condition of folic acid and tunicamycin, the invasion/migration of trophoblast cells was detected, promoting the use of the platform for drug-screening applications [29]. However, this system still has several shortcomings, such as intensive work for chip preparation, scalability of chip design, wide hydrogel scaffolds that reduce cell–cell interactions, and cell invasion taking place under the chemical gradient that occurs under both co-culture and monoculture conditions. Moreover, these systems fail to provide in-depth understanding of the basic physiology of the placenta under ideal in vivo conditions such as oxygen stress.

**Figure 1:**
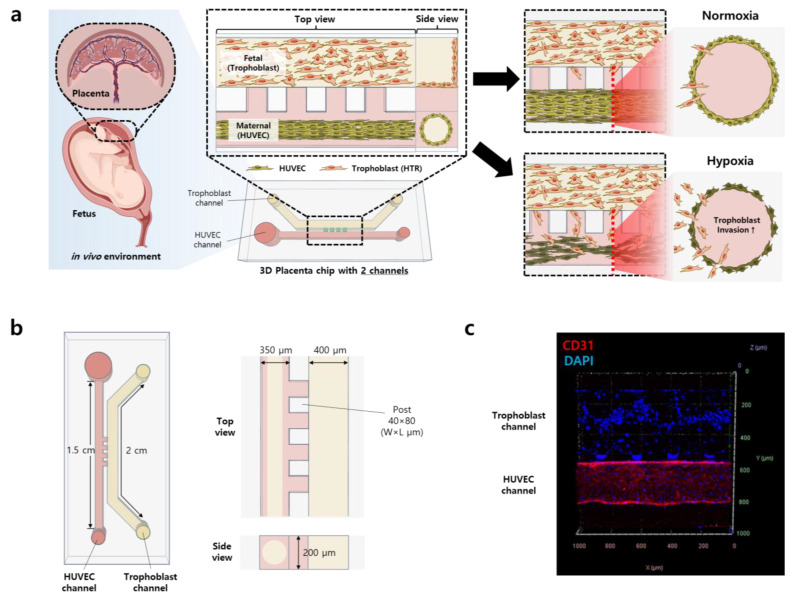
Placenta-on-a-chip: 3D microphysiological model of placental development. (**a**) Schematic figure of the 3D placenta-on-a-chip with co-cultured HUVECs and trophoblast cells (HTR-8/SVneo) for placental development. The invasion ability of trophoblasts and the function of the HUVEC vessel were evaluated under normoxic and hypoxic conditions. (**b**) Dimensions of the chip and each channel. The HUVEC channel size is 350 × 200 × 15,000 (W × H × L, μm), the trophoblast cell channel size is 400 × 200 × 20,000 (W × H × L, μm), and the post size is 40 × 200 × 80 (W × H × L, μm). Each PDMS chip size is 12 × 8 × 16 (W × H × L, mm). (**c**) Trophoblast (Top) and HUVEC (Bottom) co-cultured in the chip under the normoxic condition. The DAPI (blue) stained nucleus of the trophoblast and HUVECs. CD31 (red) is a representative marker indicating the tight junction among endothelial cells.

Therefore, to overcome the above shortcomings, we present a 3D microphysiological platform to recapitulate the physiological and biological microenvironment of the placenta and to study the effects of oxygen concentration on its developmental process. Briefly, a HUVEC vessel was formed in a microfluidic channel separated from trophoblast cells through a collagen barrier (Figure 1). The collagen acts as a barrier between both channels and provides supports to the surrounding cells. Hypoxia is generated by placing the chip in a hypoxic chamber (2% oxygen concentration) and real-time cell analysis is performed under oxygen stressed conditions. This platform can further be utilized to analyze drug effects in the early pregnancy stage for different complications as well as angiogenesis under controlled environmental conditions.

## 2. Materials and Methods

### 2.1. Cell Culture

The human first-trimester cytotrophoblast (HTR-8/SVneo) cell line was provided by Dr. Charles H. Graham (Queen’s University, Kingston, ON, Canada) and cultured in RPMI medium 1640 (Hyclone, Logan, UT, USA)) containing 5% fetal bovine serum (FBS) (GIBCO, Carlsbad, CA, USA) and 1% penicillin/streptomycin (P/S) (GIBCO, Carlsbad, CA, USA). Human umbilical vein endothelial cells (HUVECs) (ScienCell, Carlsbad, CA, USA) were cultured in endothelial cell medium (ScienCell, Carlsbad, CA, USA) containing 5% FBS, 1% endothelial cell growth supplement (ECGS) (ScienCell, Carlsbad, CA, USA), and 1% P/S.

### 2.2. Placenta-on-a-Chip Fabrication 

The master mold was fabricated by photolithography, one of the commonly used micropatterning process, and the polydimethylsiloxane (PDMS)-based microfluidic device was fabricated via soft lithography [30]. The photolithography was performed according to the manufacturer’s manual, and the process is simply described as follows. Photoresist (SU-2150, Microchem, Newton, MA, USA) is poured onto a silicon wafer and the thickness is adjusted to 200 μm using a spin coater. The film mask is aligned with an aligner (MDA-400LJ, MIDAS system, Daejeon, Republic of Korea) on a wafer that has been soft baked and irradiated with ultraviolet rays (UV) to form a fine pattern. UV is then applied to cure the micro-pattern. After passing through the post-exposure bake (PEB) process and the development process, the mold production is complete. PDMS (SYLGARD 184 A/B, DowDuPont, Midland, MI, USA) mixed at a ratio of 10:1 (wt.%) is poured into the mold. The PDMS is then placed in a desiccator to remove the gas. After complete degassing, it is cured in an oven at 80 °C for at least 3 h. The polymerized PDMS is removed from the mold and then cut, trimmed, and punched. After sterilization, the chip is attached to the slide glass by surface plasma treatment using a plasma generator (CUTE-100LF, Femtoscience, Hwaseong, Republic of Korea). The HUVEC channel size is 350 × 200 × 15,000 (W × H × L, μm), the trophoblast cell channel size is 400 × 200 × 20,000 (W × H × L, μm), and the post size is 40 × 200 × 80 (W × H × L, μm). Each PDMS chip size is 12 × 8 × 16 (W × H × L, mm).

### 2.3. Vessel Formation and Cell Seeding

For lumen fabrication, the viscous fingering method was used, which utilizes the viscosity difference between two fluids to generate a cylindrical vessel in the HUVEC channel [31]. The inlet and outlet of the HUVECs channel were punched at a diameter ratio of 1:2. Prior to loading the extracellular matrix (ECM), the device was kept at 4 °C to facilitate the proper flow of the COL-I solution into the channel. Collagen type 1 rat tail (COL-1) (Corning, New York, NY, USA) at a solution concentration of 3.5 mg/mL in cell medium was injected into the HUVEC channel, and then 5 mL COL-1 was placed at the outlet, followed by partial gelation in an incubator at 37 °C for 1 min. Subsequently, 1.5 μL of Dulbecco’s phosphate buffered saline (DPBS) (GIBCO, Carlsbad, CA, USA) was placed on the outlet and DPBS penetrated the outlet within 30 s, replacing the highly viscous COL-1 solution and creating a cylindrical vessel inside the channel. The chip was incubated again for at least 1 h for complete gelation of COL-1, followed by removal of excess COL-1 from the HUVEC channel inlet and outlets and injection of ECM media into the trophoblast cell channel. For lumen fabrication, HUVECs (3 × 10^6^ cells/mL) were injected into the COL-1 channel 4 times by rotating the chip 90° after each cell seeding followed by incubation for at least 30 min. The devices were incubated for 3 days to fabricate the full lumen structure, followed by trophoblast cell (10 × 10^6^ cells/mL) seeding in the trophoblast cells channel. The cell condition was analyzed using optical microscopy and the media was replaced every 12 h.

### 2.4. Hypoxia Generation on a Chip 

Chips were placed in an oxygen chamber (C-chamber two shelf, C274, Biospherix, Redfield, NY, USA) and cultured under a hypoxic condition (2% O2). The oxygen concentration in the chamber was adjusted by an oxygen controller (ProOx1110, BioSpherix, Redfield, NY, USA). The hypoxic culture lasted for 5 days, and the cells were analyzed every 12 h with an optical microscope.

### 2.5. Immunofluorescence Assay

For live cell invasion assay, the cells were stained with cell tracker red (C7025, Invitrogen, Carlsbad, CA, USA) and cell tracker green (C34552, Invitrogen, Carlsbad, CA, USA) in 5 µg/mL and incubated for 20 min at 37 °C. For immunostaining, Zonula occludens-1 (ZO-1) (40-2200), cluster of differentiation 31 (CD31) (MA3100), Ras homolog family member A (RhoA) (MA1-011), Ras-related C3 botulinum toxin substrate 1 (Rac1) (701942), hypoxia-inducible factor 1-alpha (HIF-1α) (MA1-516), and matrix metallopeptidase 9 (MMP-9) (MA5-15886) were purchased from Invitrogen and used following the manufacturer protocols. For nuclei staining, 4′,6-diamidino-2-phenylindole (DAPI) (D9542, Sigma-Aldrich, St. Louis, MO, USA) was used. The samples were rinsed with DPBS and fixed using 4% paraformaldehyde in DPBS for 10 min at room temperature (RT). After permeabilization using 0.1% Triton X-100 in DPBS for 15 min at RT, samples were blocked with 1% bovine serum albumin (BSA) (Sigma-Aldrich, St. Louis, MO, USA) in DPBS for 1 h at RT. Then, the samples were incubated with primary antibodies overnight at 4 °C. After washing with 0.1% BSA, the samples were incubated with secondary antibodies for 2 h at RT and nuclei were stained with 300 nM DAPI. A confocal laser scanning microscope (CLSM) (LSM 510-META, Zeiss, Oberkochen, Germany) was used for imaging and analyses.

### 2.6. Dextran Assay for HUVEC Vessel Remodeling

The HUVEC vessel remodeling was analyzed by fluorescein isothiocyanate-dextran (FITC-dextran, 70 kDa) (Sigma-Aldrich, St. Louis, MO, USA). Briefly, the FITC-dextran working solution at a concentration of 2 μg/mL was injected into the HUVEC vessel channel of the 3D chip under both hypoxic and normoxic conditions at a constant flowrate of 0.05 dynes/cm^2^. Dextran permeability was quantified by analyzing the fluorescence microscopic images at different time intervals. All experiments were performed in triplicate.

### 2.7. Apoptosis Assay

The apoptosis assay was performed using the Apoptosis/Necrosis Assay Kit (ab176749, Abcam, Boston, MA, USA). Briefly, 200 µL of assay buffer were prepared by mixing 1 µL of 7-aminoactinomycin D (7-AAD, 200X), 1 µL of CytoCalcein violet 450 (200X), and 2 µL of (100X) Apopxin green indicator mixed solution. The buffer was injected into the channel and incubated for 1 h at RT. Cell analysis was performed using optical microscopy.

### 2.8. Statistical Analysis 

Results are expressed as mean ± standard error of the mean (S.E.M.). Statistical significance was evaluated using a two-tailed Student’s *t*-test. Each experiment was replicated at least three times. For fluorescence images, quantification was performed by image processing using ImageJ software (1.52 ver.).

## 3. Results and Discussion

### 3.1. Construction of 3D Placenta-on-a-Chip

**Figure 2:**
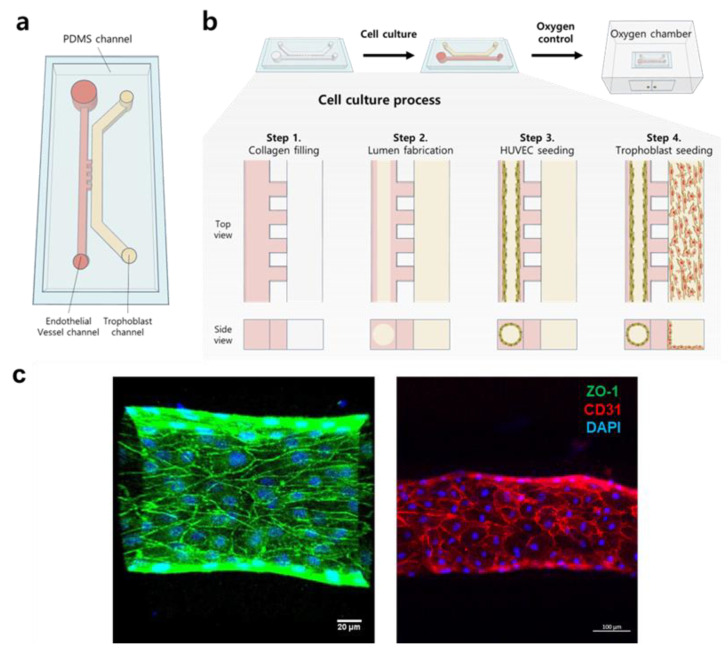
Fabrication scheme of placenta-on-chip and HUVEC vessel characterization. (**a**) Schematic design of the 3D placenta-on-a-chip with the HUVEC channel and trophoblast channel separated by 50 μm-sized posts. (**b**) Experimental scheme: The upper figures represent a top view, and the lower figure represents a cross-section view. Step 1: COL-1 is injected through the HUVEC channel, and the gel also occupies the post area due to the high surface tension in the chip. Step 2: Lumen patterning is performed after semi-polymerization of the COL-1, followed by gelation. Step 3: HUVECs are seeded, the medium is replaced every 12 h, and the cells are incubated for 3 days until they are fully grown. Step 4: Trophoblast cell seeding and culture in a normoxic/hypoxic chamber. (**c**) Confocal 3D images of the HUVEC vessel. Green, red, and blue show ZO-1, CD31, and DAPI staining, respectively. Left: front view of the HUVEC vessel tight junction (green) and cell nuclei (blue), Right: front view of the HUVEC vessel with capillary-like structure formation using CD31 (red) and cell nuclei (blue). Confluent HUVECs formed in the circular lumen. ZO-1 and CD31 staining demonstrate the tight junctions of HUVECs after 3 days of culture. Scale bars, 20 μm (left and middle) and 100 μm (right).

The design of the 3D placenta-on-a-chip consists of two channels separated by a small constriction that maintains the minimum distance between the two cells and provides the barrier functions shown in Figure 2a. HUVECs were cultured inside a cylindrical collagen to form a microvessel by the viscous fingering method [31] to recapitulate a spiral artery structure (Figure 2c) [32]. Briefly, the HUVEC channel was first filled with collagen (COL-1). Subsequently, HUVECs were lined on the COL-1 and cultured for 3 continuous days to form a vessel structure following the trophoblast cell culturing in the trophoblast channel. Tight junctions of HUVECs were identified by the representative HUVEC cell markers CD31 and ZO-1 (Figure 2c). ZO-1 plays a key role in the regulation of tension in the HUVEC cell barrier [33], and CD31, also known as PECAM-1, is found in high concentrations in HUVEC cell intercellular junctions, where it acts in the maintenance of HUVEC cell junctional integrity [34]. To confirm the endothelial barrier function of HUVECs, immunostaining assays were performed, and three-dimensional z-stack images were acquired with a confocal microscope. The results confirmed the tight junction and cylindrical vessel shape with reticular CD31 (red) and ZO-1 (green) fluorescence between HUVECs throughout the whole HUVEC channel. The well-formed cylindrical structure with a hollow interior, like in vivo blood vessels, was also confirmed (Figure S1). The straightforward design of our microfluidic device allows for easier replication of HUVEC vessels, facilitating 3D analysis of placental remodeling compared to what has been previously reported, with more intricate microfluidic platforms. 

### 3.2. Hypoxic Environment Promotes the Invasion Ability of Trophoblasts

**Figure 3:**
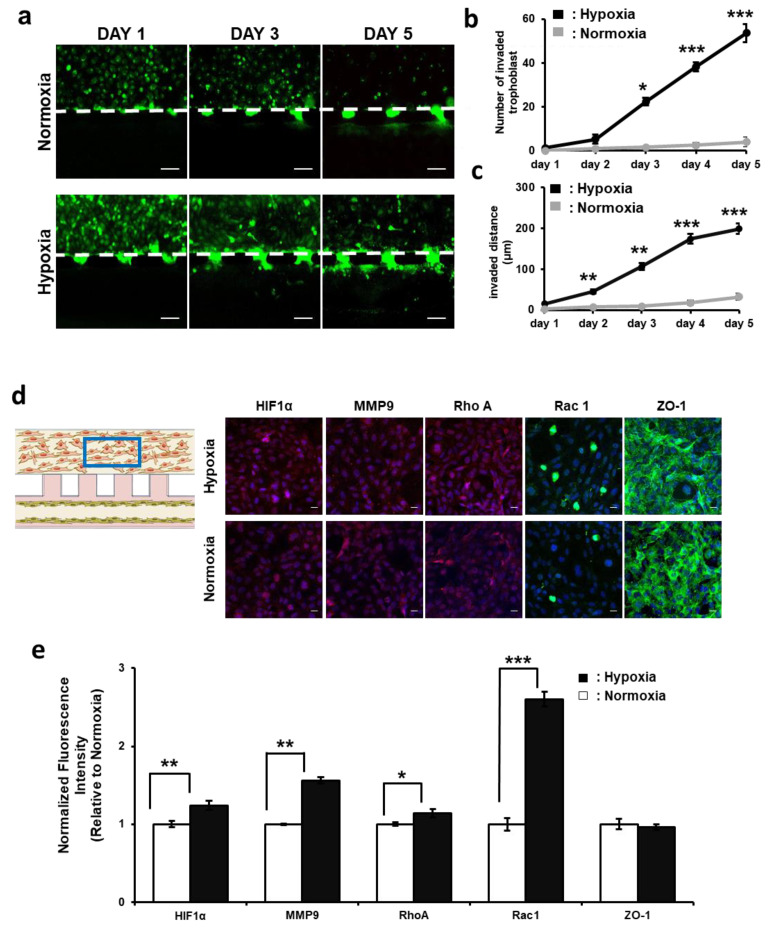
Trophoblast cell invasion under hypoxic and normoxic conditions (**a**) Time-lapse images of trophoblast cells under normoxic and hypoxic conditions at days 1, 3, and 5. Trophoblast cells were stained with cell tracker green. Because HUVECs were not fluorescently labeled in the chip, they were not visible in the images. Scale bar = 100 μm. (**b**) Quantification of the number of invaded trophoblast cells. (**c**) Distance of trophoblast cell invasion into the HUVEC vessel channel. (**d**) Immunofluorescence images of trophoblast cells. HIF-1α-, MMP-9-, Rac1-, RhoA-, and ZO-1-stained images under hypoxic and normoxic conditions. Scale bars, 20 μm. (**e**) Quantifications of protein expressions. (* indicates the difference between normoxic and hypoxic. * *p* < 0.05, ** *p* < 0.01, *** *p* < 0.001).

To compare the invasion ability of trophoblast cells under hypoxic and normoxic conditions, the trophoblast cells were dyed with cell tracker green and analyzed every 24 h in both normoxic (21% O_2_) and hypoxic (2% O_2_) conditions (Figure 3a). Under the normoxic condition, no or very little invasion of trophoblast cells was observed over a period of 5 days, whereas under the hypoxic condition, aggressive invasion of trophoblast cells occurred after day 3. Furthermore, this trophoblast invasion ability further increased over the span of 5 days, and the number and distance of the invaded cells were upregulated into the HUVEC vessel. The number of invaded cells was counted under both hypoxic and normoxic conditions over the period of 5 days, and it was found that the number of invaded trophoblast cells in the hypoxic condition was 13 times higher than the number of cells in the normoxic condition (Figure 3b). In addition, the invasion distance under the hypoxic condition showed a deeper invasion of the trophoblast cells into the HUVEC vessel compared to the cells under the normoxic condition (Figure 3c). It was observed that the difference between the number of invaded cells was initially very small on day 1 under both hypoxic and normoxic conditions and increased exponentially from day 2 to day 5. In contrast, the invaded distance increased continuously from day 1 to day 5. These results show that hypoxia upregulates the invasion ability of trophoblasts. 

Furthermore, an immunofluorescence assay was performed to analyze the protein expressions associated with trophoblast invasion under the hypoxic condition. HUVECs and trophoblast cells were co-cultured under hypoxic and normoxic conditions for five days, and on day 5, immunofluorescence assay was performed to analyze the five different protein expressions: HIF-1α, MMP-9, RhoA, Rac1, and ZO-1 (Figure 3d). All proteins were observed in the trophoblast cell channel, and the fluorescence intensity was normalized to the area of the trophoblast cell channel. HIF-1α fluorescence intensity was increased by 24% in the hypoxic condition compared to the normoxic condition (Figure 3e). Similarly, MMP-9 fluorescence intensity increased by 36%, RhoA fluorescence intensity increased by 16%, and Rac1 fluorescence intensity increased by 56% in the hypoxic condition compared to the normoxic condition. These results show that hypoxia activates and upregulates HIF-1α expression, which is the critical factor for cell survival under a low-oxygen-concentration environment and a key regulator in the upregulation of other related genes involved in cell migration/invasion and extracellular matrix remodeling. Under hypoxia, increased expression of HIF-1α activates transcription of the MMP-9 protein, which is primarily responsible for extracellular matrix degradation and promotes trophoblast invasion through the surrounding cell layer [35,36,37]. Furthermore, Rac1 is another key modulator of trophoblast invasion behavior, and the increased expression of Rac1 explains the deeper invasion of trophoblast cells into the HUVEC vessel, as it promotes cytoskeletal rearrangement and actin polymerization, which enhances cell invasion by allowing the trophoblast cell to detach from the extracellular matrix and thus penetrate deeper into the HUVEC vessel. In addition, this invasion is complemented by the upregulation of RhoA, which modulates the integrity of cell tight junctions and influences the trophoblast invasion. In contrast, no significant change in basement membrane protein (ZO-1) expression was observed under the hypoxic condition [33].

### 3.3. HUVEC Vessel Remodeling by Trophoblast Invasion 

**Figure 4:**
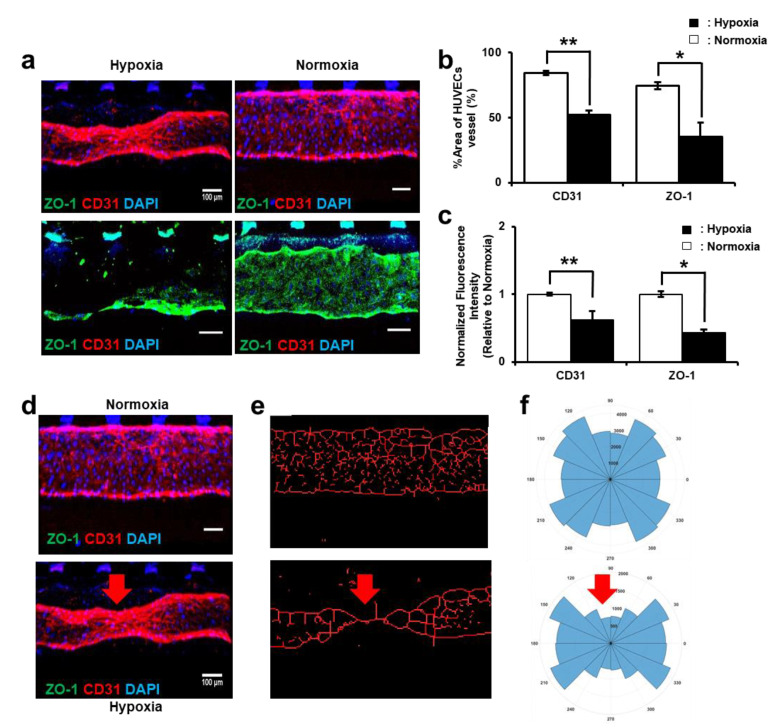
Vascular remodeling of HUVECs co-cultured with trophoblast cells under hypoxic condition. (**a**) Immunostaining of ZO-1 (green), CD31 (red), and DAPI (blue) expression by HUVECs cultured after 5 days of co-culture. Vessel function was evaluated in both hypoxic and normoxic conditions. Scale bars, 100 μm. (**b**) Quantification of % area of the HUVEC vessels. (**c**) Normalized fluorescence intensity of ZO-1 and CD31. (**d**) Immunostaining images of HUVEC vessel in normoxic and hypoxic conditions. (**e**) Cell centerline analysis of the HUVEC vessel of corresponding normoxia and hypoxia images. Cell centerline analysis shows the damaged morphology in hypoxia. (**f**) Histogram plot analysis of the HUVEC vessel in normoxic and hypoxic conditions. The distribution shows that the deformation of the vessel is more prominent at the trophoblast invasion point (shown in red arrow). (* *p* < 0.05, ** *p* < 0.01).

HUVEC vessel remodeling was analyzed in co-culture conditions under normoxic and hypoxic conditions on day 8. CD31 and ZO-1 showed a prominent HUVEC deformation under the hypoxic condition compared to the normoxic condition. In particular, a severe deformation was detected near the post region (shown in white in Figure 3a), where the trophoblast invasion started and weakened the cell–cell junctions of the HUVEC vessel. Under the normoxic condition, the linear vessel shape was relatively well preserved and the tight junctions between cells were also confirmed (Figure 4a). Additionally, the % area of the HUVEC vessel was calculated by measuring the intensity level of CD31 and ZO-1 for hypoxic and normoxic conditions using ImageJ software. In the hypoxic condition, the CD31 % area of HUVEC vessel decreased by 38% and the ZO-1 % area of the HUVEC vessel decreased by 52% compared to the normoxic condition (Figure 4b). Similarly, when comparing the relative fluorescence intensity, the CD31 fluorescence intensity decreased from the normoxic to the hypoxic condition by 62% and the ZO-1 fluorescence intensity decreased by 43% from the normoxic to hypoxic condition (Figure 4c). These results confirm that the downregulation of CD31 fluorescence intensity during hypoxia is due to a potential remodeling of the HUVEC vessel, which is associated with changes in the HUVEC cell junctions and cell–cell adhesion properties. As trophoblast cells invade the HUVEC vessel, they may induce alterations in the HUVEC cell layer to facilitate their migration and penetration into the underlying tissue. Similarly, downregulation of ZO-1 indicates the disruption of HUVEC vessel tight junction by the trophoblast invasion. To further analyze the HUVEC vessel morphology under hypoxic and normoxic conditions, we performed cell centerline analysis (Figure 4d). The HUVEC vessel in the hypoxic condition showed distorted vessel morphology compared to the normoxic environment (Figure 4e), as a reduction in cell centerlines was observed. This decrease in cell centerlines suggests that trophoblast invasion affects the integrity of HUVEC vessels by damaging the cell–cell tight junction, which is responsible for the formation of HUVEC vessel walls. Furthermore, the histogram plot also confirmed the distortion of the HUVEC vessel under the hypoxic condition, as the vessel shrank at the point of trophoblast cell invasion (Figure 4f). This indicates that the distortion was more pronounced in one direction, which was the direction of trophoblast cell invasion at the specific points. These results confirm that the invasion of trophoblast cells under hypoxic conditions has a clear effect on vessel morphology. Although spiral arteries expand in vivo [34,38], our study showed shrinkage of the HUVEC vessel mainly due to the direction of trophoblast invasion and that blood perfusion was difficult to fully realize on the chip, as reported in other studies [39,40]. However, our results show the same tight junction weakening and morphological changes in the HUVEC vessels, similar to what has previously been reported in in vivo systems [41,42,43]. Moreover, this HUVEC vessel structural distortion suggests that trophoblast cell invasion remodels the vessel by interdigitating between the HUVEC cells and coexisting on the vessel wall [44,45,46]. One of the reasons for the enhanced HUVEC vascular remodeling under hypoxia is that trophoblast cells rearrange and release the extracellular matrix digestion proteins, which, together with HUVECs, promote invasion by creating more voids in the ECM space [47,48]. This was further supported by analysis of the decrease in ZO-1, a cell tight junction marker, following trophoblast invasion.

**Figure 5:**
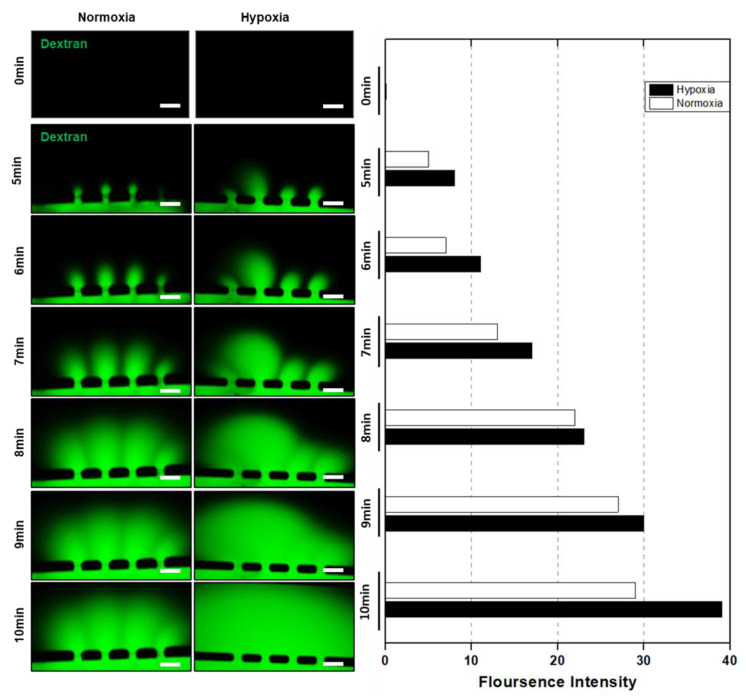
Vessel permeability analysis: time-lapse images of permeability assay with 70 kDa dextran. The dextran was injected into the HUVEC channel, and fluorescence images were captured at different time intervals. The fluorescence intensity was analyzed using ImageJ analysis for both normoxic and hypoxic conditions. Scale bars = 100 μm.

In addition, a dextran permeability assay was performed to analyze the effect of structural distortion on vascular permeability (Figure 5). After 5 min of injection, the FITC-dextran solution began to flow into the trophoblast cell channel, indicating permeability and exchange of fluorescently labeled dextran between the vessel and the trophoblast cells. A rapid diffusion of dextran into the trophoblast cell channel was observed from 5 to 7 min, and fluorescence images show that the dextran diffusion in the hypoxic condition was significantly higher than in the normoxic condition. Furthermore, the dextran diffusion from 7 to 10 min showed a higher signal of dextran in the hypoxic condition, which confirms the HUVEC vessel deformation upon trophoblast cell invasion.

### 3.4. HUVEC Cell Apoptosis Induction by Trophoblast Invasion

**Figure 6:**
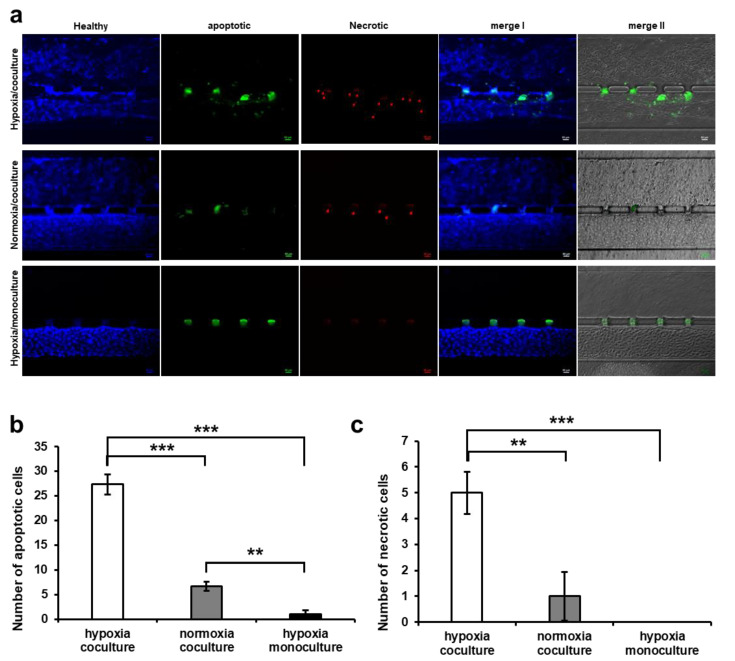
Apoptosis assay at the placenta-on-a-chip. (**a**) Fluorescence images of apoptosis assay. Healthy cells (blue, stained by cytocalcein violet 450), apoptotic cells (green, stained by apopsin green), and last stage of apoptotic and necrotic cells (red, stained by 7-aminoactinomycin D, the arrows indicate the presence of necrotic cells in the image) were identified. Images were taken under three different conditions: hypoxic/co-culture, normoxic/co-culture, and hypoxic/HUVEC monoculture. Merge 1 refers to a combination of healthy images and apoptotic images. Merge 1 refers to a combination of brightfield images and apoptotic images. Scale bars, 50 μm. (**b**) The quantification of the number of apoptotic cells. (**c**) The number of necrotic cells. (** *p* < 0.01, *** *p* < 0.001). The apoptosis assay was performed using the Apoptosis/Necrosis Assay Kit (ab176749, abcam, Boston, MA, USA).

To investigate the state of HUVECs after trophoblast invasion and to ensure that hypoxia alone did not adversely affect the morphology of HUVEC vasculature, an apoptosis assay was performed under three different experimental conditions: (i) HUVEC monoculture in the hypoxic condition, (ii) co-culture with trophoblast cells in the hypoxic condition, and (iii) co-culture with trophoblast cells in the normoxic condition (Figure 6a). Three different fluorescence colors were used to analyze the state of the cells: blue (healthy cell), green (initial apoptosis), and red (necrosis, last stage of apoptosis). In the hypoxic condition, green fluorescence was upregulated in the HUVEC channel in the co-culture condition (iii) compared to co-culture with trophoblast cells in the hypoxic condition (ii) and HUVEC monoculture in the hypoxic condition (i). On the other hand, red fluorescence (apoptosis marker) was rarely expressed in the HUVEC monoculture in the hypoxic (i) condition (Figure 6b). The apoptosis of HUVECs was significant at a site where the invasion of trophoblast cells was active and at the site where the morphological distortion occurred in the HUVEC vessel in conditions (ii) and (iii) (merge I and merge II images in Figure 6a). In addition, a higher level of apoptotic cells was observed in co-culture with trophoblast in the normoxic condition than in monoculture in the hypoxic condition. This suggests that co-culture has more influence on the apoptosis of HUVECs than the oxygen concentration. Similarly, the necrotic signal was 3.75 times higher in the hypoxic co-culture (ii) than in the normoxic co-culture (iii) or hypoxic monoculture (i) because no fluorescent signal was detected (Figure 6c). These results show that hypoxia alone does not affect HUVECs, but the invasion of trophoblast cells induces apoptosis, which is upregulated by the direct contact between HUVECs and trophoblast cells. This can be further explained by the fact that in the co-culture condition, the trophoblast cell invasion replaced the HUVEC vascular lining and impaired the cell tight junction, which led to HUVEC cell apoptosis. The increased apoptotic number of cells in the co-culture hypoxic environment indicates that oxygen stress alone has no effect on remodeling or angiogenesis, but trophoblast cells in hypoxia secrete migration/invasion proteins including MMPs, RhoA, and Rac1, which upregulate the invasion by deforming the extracellular matrix and HUVEC cell junction. These results are consistent with the previously reported in vivo studies that trophoblast invasion in placental vessels is promoted by displacing HUVEC cells and secreting various growth factors that affect HUVEC cell survival [49].

## 4. Conclusions

In conclusion, we have established a 3D microphysiological platform that allows direct insight into placental remodeling under oxygen-stressed conditions. The results suggest that hypoxia is the key driving factor for trophoblast invasion into the HUVEC vessel, which leads to HUVEC tight junction deformation and consequently induces cell apoptosis. Therefore, we can infer that this platform replicates in vivo conditions and can be utilized to analyze different strategies for managing pregnancy-related issues. Although our system successfully mimicked the microphysiological conditions of placental remodeling under hypoxia, our work can be extended to model systems to analyze the effects of other factors such as blood flow shear stress through the perfusion system, nanoparticles, complex ECM composition, or intercellular nutrient exchange effects on HUVEC remodeling, as well as the effects of various drugs.

## Data Availability

The datasets used and/or analyzed during the current study are available from the corresponding author on reasonable request.

## Supplementary Materials

The following supporting information can be downloaded at: https://www.mdpi.com/article/10.3390/biomimetics9050289/s1, Table S1: Summarization of different experimental techniques employed to study placental development.; Figure S1: Confocal 3D images with cross-sectional view of the HUVEC vessel.
