# Supplementary material for: A Microphysiological Model to Mimic the Placental Remodeling during Early Stage of Pregnancy under Hypoxia-Induced Trophoblast Invasion
Source: Biomimetics (Basel). 2024 May 12;9(5):289. doi: 10.3390/biomimetics9050289 (PMC11118815; doi:10.3390/biomimetics9050289)
Supplement: Supplementary file 1 [file biomimetics-09-00289-s001.zip › biomimetics-2945262-supplementary.pdf]

Supporting Information for

A Microphysiological Model to Mimic the Placental Remodeling during Early Stage of Pregnancy under Hypoxia Induced Trophoblast Invasion

Seorin Jeong[1], Ahmed Fuwad[1], Sunhee Yoon[2], Tae-Joon Jeon [2,3,4,†], Sun Min Kim[1,2,3,†]

[1] Department of Mechanical Engineering, Inha university, 100, Inha-ro, Michuhol-gu, Incheon 22212, Republic of Korea

[2] Department of Biological Science and Bioengineering, Inha University, 100, Inha-ro, Michuhol-gu, Incheon 22212, Republic of Korea

[3] Biohybrid Systems Research Center, Inha University, 100, Inha-ro, Michuhol-gu, Incheon 22212, Korea

[4] Department of Biological Engineering, Inha university, 100, Inha-ro, Michuhol-gu, Incheon 22212, Republic of Korea

Corresponding Authors:

Tae-Joon Jeon: tjjeon@inha.ac.kr

Sun Min Kim: sunmk@inha.ac.kr

Table S1 Summarization of different experimental techniques employed to study the placenta development.

Model	Benefit	Limitations	Application	Ref
**2D culture**	- Sustainable cell culture<br>- Maintain in vitro characteristics.<br>- Cell-cell interaction studies<br>- Enable signal pathway studies	- Cell type limitations<br>- Challenging to study between cells and membrane<br>- No physiological relevance	Novel platform / Functional study	[1–9]
			Toxicity/drug test	[10–12]
			Substance transfer	[13,14]
**Animal model**	- Longitudinal sample available<br>- Anatomical similarities	- Expensive to maintain<br>- Difficult handling<br>- Difficult to construct suitable animal model	Novel platform / Functional study	[15,16]
			Toxicity/drug test	[17–20]
			Substance transfer	[20,21]
**Placenta -on-a -chip**	- Mimics in vivo signaling.<br>- Correct tissue organization<br>- Enables coculture conditions	- Hard to acquire samples for further analysis<br>- Small cell number leads to limited phenotypic assay<br>- Difficult to culture cells on both sides the membrane	Novel platform / Functional study	[22–26]
			Toxicity/drug test	[27,28]
			Substance transfer	[29–32]

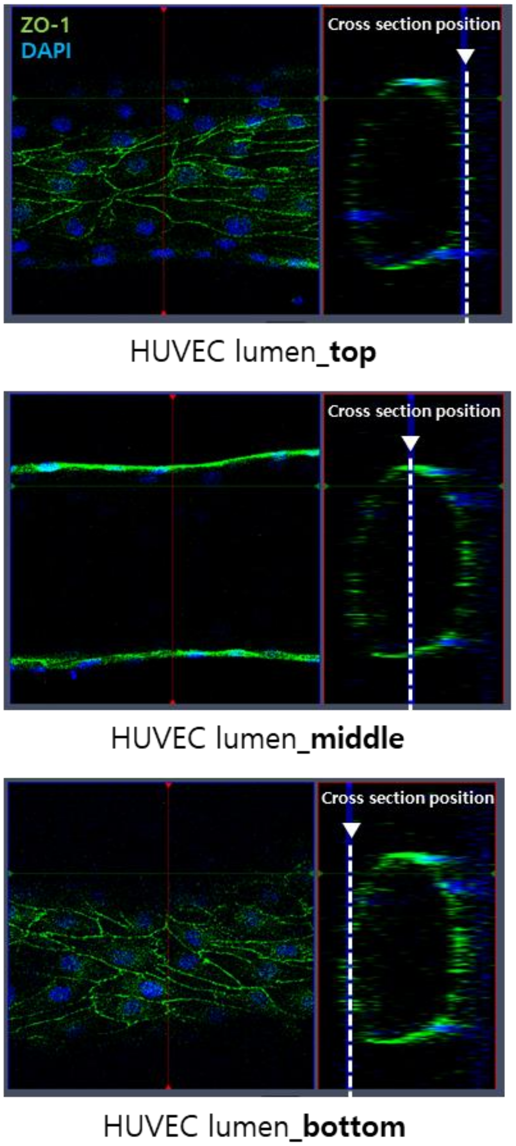

Figure S1. Confocal 3D images with cross sectional view of HUVECs vessel. Green and blue show ZO-1 and DAPI staining, respectively. Well-formed cylindrical structure with a hollow interior, like *in vivo* blood vessel was confirmed.

References

1. Rytting, E.; Cartwright; Poulsen; Nielsen; Pojana; Knudsen; Saunders In Vitro Placental Model Optimization for Nanoparticle Transport Studies. *International Journal of Nanomedicine* **2012**, *7*, 497

2. Duzyj, C.M.; Barnea, E.R.; Li, M.; Huang, S.J.; Krikun, G.; Paidas, M.J. Preimplantation Factor Promotes First Trimester Trophoblast Invasion. *American Journal of Obstetrics and Gynecology* **2010**, *203*, 402.e1-402.e4

3. Nathenson, M.J.; Barysauskas, C.M.; Nathenson, R.A.; Regine, W.F.; Hanna, N.; Sausville, E. Surgical Resection for Recurrent Retroperitoneal Leiomyosarcoma and Liposarcoma. *World Journal of Surgical Oncology* **2018**, *16*, 1–11

4. Müller, E.K.; Gräfe, C.; Wiekhorst, F.; Bergemann, C.; Weidner, A.; Dutz, S.; Clement, J.H. Magnetic Nanoparticles Interact and Pass an in Vitro Co-Culture Blood-Placenta Barrier Model. *Nanomaterials* **2018**, *8*, 108

5. Nishiguchi, A.; Gilmore, C.; Sood, A.; Matsusaki, M.; Collett, G.; Tannetta, D.; Sargent, I.L.; McGarvey, J.; Halemani, N.D.; Hanley, J.; et al. In Vitro Placenta Barrier Model Using Primary Human Trophoblasts, Underlying Connective Tissue and Vascular Endothelium. *Biomaterials* **2019**, *192*, 140–148

6. Blanco, O.; Leno-Durán, E.; Morales, J.C.; Olivares, E.G.; Ruiz-Ruiz, C. Human Decidual Stromal Cells Protect Lymphocytes from Apoptosis. *Placenta* **2009**, *30*, 677–685

7. Tan, L.; Chen, Z.; Sun, F.; Zhou, Z.; Zhang, B.; Wang, B.; Chen, J.; Li, M.; Xiao, T.; Neuman, R.I.; et al. Placental Trophoblast-Specific Overexpression of Chemerin Induces Preeclampsia-like Symptoms. *Clinical Science* **2022**, *136*

8. Wong, M.K.; Li, E.W.; Adam, M.; Selvaganapathy, P.R.; Raha, S. Establishment of an in Vitro Placental Barrier Model Cultured under Physiologically Relevant Oxygen Levels. *Molecular human reproduction* **2020**, *26*, 353–365

9. Walsh, S.W. Plasma from Preeclamptic Women Stimulates Transendothelial Migration of Neutrophils. *Reproductive Sciences* **2009**, *16*, 320–325

10. da Costa Garcia Filho, F.; da Luz, F.S.; Nascimento, L.F.C.; Satyanarayana, K.G.; Drelich, J.W.; Monteiro, S.N. Mechanical Properties of Boehmeria Nivea Natural Fabric Reinforced Epoxy Matrix Composite Prepared by Vacuum-Assisted Resin Infusion Molding. *Polymers* **2020**, *12*, 1311

11. Tannetta, D.S.; Sargent, I.L.; Linton, E.A.; Redman, C.W.G. Vitamins C and E Inhibit Apoptosis of Cultured Human Term Placenta Trophoblast. *Placenta* **2008**, *29*, 680–690

12. Orendi, K.; Gauster, M.; Moser, G.; Meiri, H.; Huppertz, B. Effects of Vitamins C and E, Acetylsalicylic Acid and Heparin on Fusion, Beta-HCG and PP13 Expression in BeWo Cells. *Placenta* **2010**, *31*, 431–438

13. Huang, X.; Lüthi, M.; Ontsouka, E.C.; Kallol, S.; Baumann, M.U.; Surbek, D. V.; Albrecht, C. Establishment of a Confluent Monolayer Model with Human Primary Trophoblast Cells: Novel Insights into Placental Glucose Transport. *Molecular Human Reproduction* **2016**, *22*, 442–456

14. Aengenheister, L.; Keevend, K.; Muoth, C.; Schönenberger, R.; Diener, L.; Wick, P.; Buerki-Thurnherr, T. An Advanced Human in Vitro Co-Culture Model for Translocation Studies across the Placental Barrier. *Scientific Reports* **2018**, *8*, 5388

15. Barry, J.S.; Anthony, R. V. The Pregnant Sheep as a Model for Human Pregnancy. *Theriogenology* **2008**, *69*, 55–67

16. Thompson, L.P.; Pence, L.; Pinkas, G.; Song, H.; Telugu, B.P. Placental Hypoxia during Early Pregnancy Causes Maternal Hypertension and Placental Insufficiency in the Hypoxic Guinea Pig Model. *Biology of Reproduction* **2016**, *95*, 1–10

17. Zhang, B.; Tan, L.; Yu, Y.; Wang, B.; Chen, Z.; Han, J.; Li, M.; Chen, J.; Xiao, T.; Ambati, B.K.; et al. Placenta-Specific Drug Delivery by Trophoblast-Targeted Nanoparticles in Mice. *Theranostics* **2018**, *8*, 2765–2781

18. Andersen, M.H.G.; Zuri, G.; Knudsen, L.E.; Mathiesen, L. Placental Transport of Parabens Studied Using an Ex-Vivo Human Perfusion Model. *Placenta* **2021**, *115*, 121–128

19. Smith, J.A.; Gaikwad, A.; Mosley, S.; Coffer, L.; Cegelski, J.; Alcorn, J.L.; Ramin, S.M.; Refuerzo, J.S. Utilization of an Ex Vivo Human Placental Perfusion Model to Predict Potential Fetal Exposure to Carboplatin during Pregnancy. *American Journal of Obstetrics and Gynecology* **2014**, *210*, 275.e1-275.e9

20. Durhan, G.; Ünverdi, H.; Deveci, C.; Büyükşireci, M.; Karakaya, J.; Değirmenci, T.; Bayrak, A.; Koşar, P.; Hücümenoğlu, S.; Ergün, Y. Placental Elasticity and Histopathological Findings in Normal and Intra-Uterine Growth Restriction Pregnancies Assessed with Strain Elastography in Ex Vivo Placenta. *Ultrasound in Medicine and Biology* **2017**, *43*, 111–118

21. Berveiller, P.; Mir, O.; Vinot, C.; Bonati, C.; Duchene, P.; Giraud, C.; Gil, S.; Treluyer, J.M. Transplacental Transfer of Oseltamivir and Its Metabolite Using the Human Perfused Placental Cotyledon Model. *American Journal of Obstetrics and Gynecology* **2012**, *206*

22. Pu, Y.; Gingrich, J.; Veiga-Lopez, A. A 3-Dimensional Microfluidic Platform for Modeling Human Extravillous Trophoblast Invasion and Toxicological Screening. *Lab on a Chip* **2021**, *21*, 546–557

23. Mosavati, B.; Oleinikov, A. V.; Du, E. Development of an Organ-on-a-Chip-Device for Study of Placental Pathologies. *International Journal of Molecular Sciences* **2020**, *21*, 1–12

24. Park, J.Y.; Mani, S.; Clair, G.; Olson, H.M.; Paurus, V.L.; Ansong, C.K.; Blundell, C.; Young, R.; Kanter, J.; Gordon, S.; et al. A Microphysiological Model of Human Trophoblast Invasion during Implantation. *Nature Communications* **2022**, *13*

25. Richardson, L.S.; Kim, S.; Han, A.; Menon, R. Modeling Ascending Infection with a Feto-Maternal Interface Organ-on-Chip. *Lab on a Chip* **2020**, *20*, 4486–4501

26. Barrett, D.W.; John, R.K.; Thrasivoulou, C.; Mata, A.; Deprest, J.A.; Becker, D.L.; David, A.L.; Chowdhury, T.T. Targeting Mechanotransduction Mechanisms and Tissue Weakening Signals in the Human Amniotic Membrane. *Scientific Reports* **2019**, *9*, 1–11

27. Radnaa, E.; Richardson, L.S.; Sheller-Miller, S.; Baljinnyam, T.; De Castro Silva, M.; Kumar Kammala, A.; Urrabaz-Garza, R.; Kechichian, T.; Kim, S.; Han, A.; et al. Extracellular Vesicle

Mediated Feto-Maternal HMGB1 Signaling Induces Preterm Birth. *Lab on a Chip* **2021**, *21*, 1956–1973

28. Yu, F.; Hunziker, W.; Choudhury, D. Engineering Microfluidic Organoid-on-a-Chip Platforms. *Micromachines* **2019**, *10*, 165

29. Richardson, L.; Gnecco, J.; Ding, T.; Osteen, K.; Rogers, L.M.; Aronoff, D.M.; Menon, R. Fetal Membrane Organ-On-Chip: An Innovative Approach to Study Cellular Interactions. *Reproductive Sciences* **2020**, *27*, 1562–1569

30. Boos, J.A.; Misun, P.M.; Brunoldi, G.; Furer, L.A.; Aengenheister, L.; Modena, M.; Rousset, N.; Buerki-Thurnherr, T.; Hierlemann, A. Microfluidic Co-Culture Platform to Recapitulate the Maternal–Placental–Embryonic Axis. *Advanced Biology* **2021**, *5*, 2100609

31. Kreuder, A.E.; Bolaños-Rosales, A.; Palmer, C.; Thomas, A.; Geiger, M.A.; Lam, T.; Amler, A.K.; Markert, U.R.; Lauster, R.; Kloke, L. Inspired by the Human Placenta: A Novel 3D Bioprinted Membrane System to Create Barrier Models. *Scientific Reports* **2020**, *10*, 1–14

32. Kuo, C.Y.; Shevchuk, M.; Opfermann, J.; Guo, T.; Santoro, M.; Fisher, J.P.; Kim, P.C.W. Trophoblast–Endothelium Signaling Involves Angiogenesis and Apoptosis in a Dynamic Bioprinted Placenta Model. *Biotechnology and Bioengineering* **2019**, *116*, 181–192
